# Simulation of gene family histories

**DOI:** 10.1186/1471-2105-15-S3-A8

**Published:** 2014-02-11

**Authors:** Maribel Hernandez-Rosales, Nicolas Wieseke, Marc Hellmuth, Peter F Stadler

**Affiliations:** 1 Max-Planck-Institute for Mathematics in the Sciences, Inselstr. 22, D-04103 Leipzig, Germany; 2 Department of Computer Science, Univ. Leipzig, Härtelstr. 16-18, D-04107 Leipzig, Germany

## Background

The way gene families and genomes evolve can be understood in detail only when the location of gene duplication episodes in the tree of life can be deciphered. Since most genes belong to larger gene families, the analysis of the gene family histories thus plays an important role in the study of genome evolution. Empirically, one frequently observes that the tree that describes the evolution of species, the species tree, is inconsistent with the tree that is obtained from a group of genes of a gene family (the gene tree). Goodman et al. deduced that this inconsistency might be the result of mistaking paralogs for orthologs. Orthologous genes refer to copies of genes that reveal the phylogeny of species, while paralogous genes have been created by duplication events. Phylogeny reconstruction can help to understand how gene families evolved and to identify the chronology of duplications within a gene family of a single species. Several software tools, including GeneTree, DupTree, NOTUNG, and AUGIST have been developed for this task. There is, however, lack of both test data and evaluation procedures to test, compare, and benchmark their performance and results.

## Results

We present here a simulation environment designed to generate large gene families with complex duplication histories on which reconstruction algorithms can be tested and software tools can be benchmarked.

The simulation of gene family histories starts with the generation of species trees. Within these rooted bifurcating trees the nodes represent species and edges their relation. Specifically, internal nodes represent ancient species whereas leaf nodes represent extant species. Given a number of species N, we generate a random tree T under the Age Model described in Keller-Schmidt et al. This model starts with a rooted tree with two leaves. In an iterative process one of the leaves is selected and two new leaves are attached to it until the tree has N leaves. This model makes use of the idea that the longer a leaf has not been involved in a speciation, the less likely it will be in the future.

**Figure 1:**
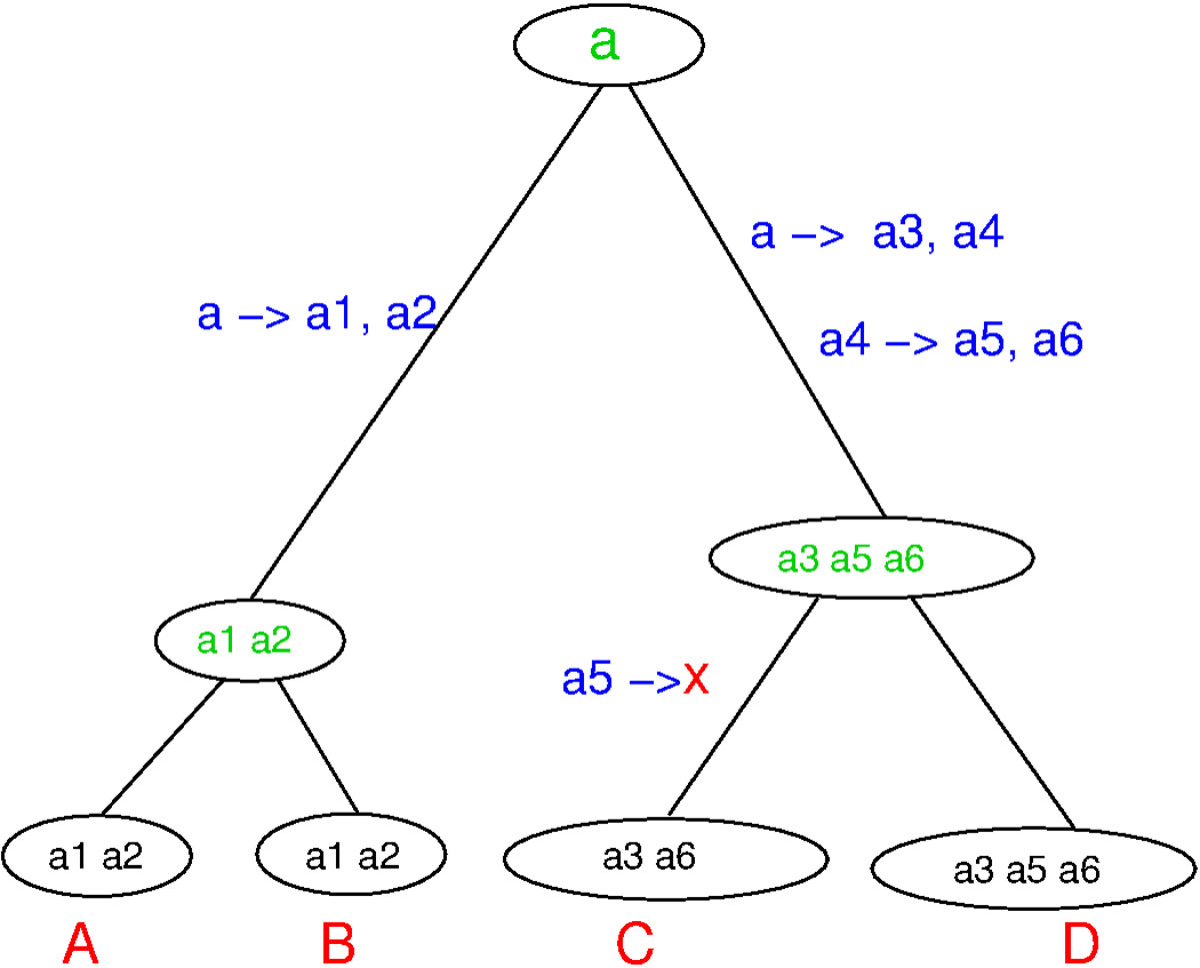
A one-gene family history: from a node parent to a node child, there could be duplications and losses of genes.

**Figure 2:**
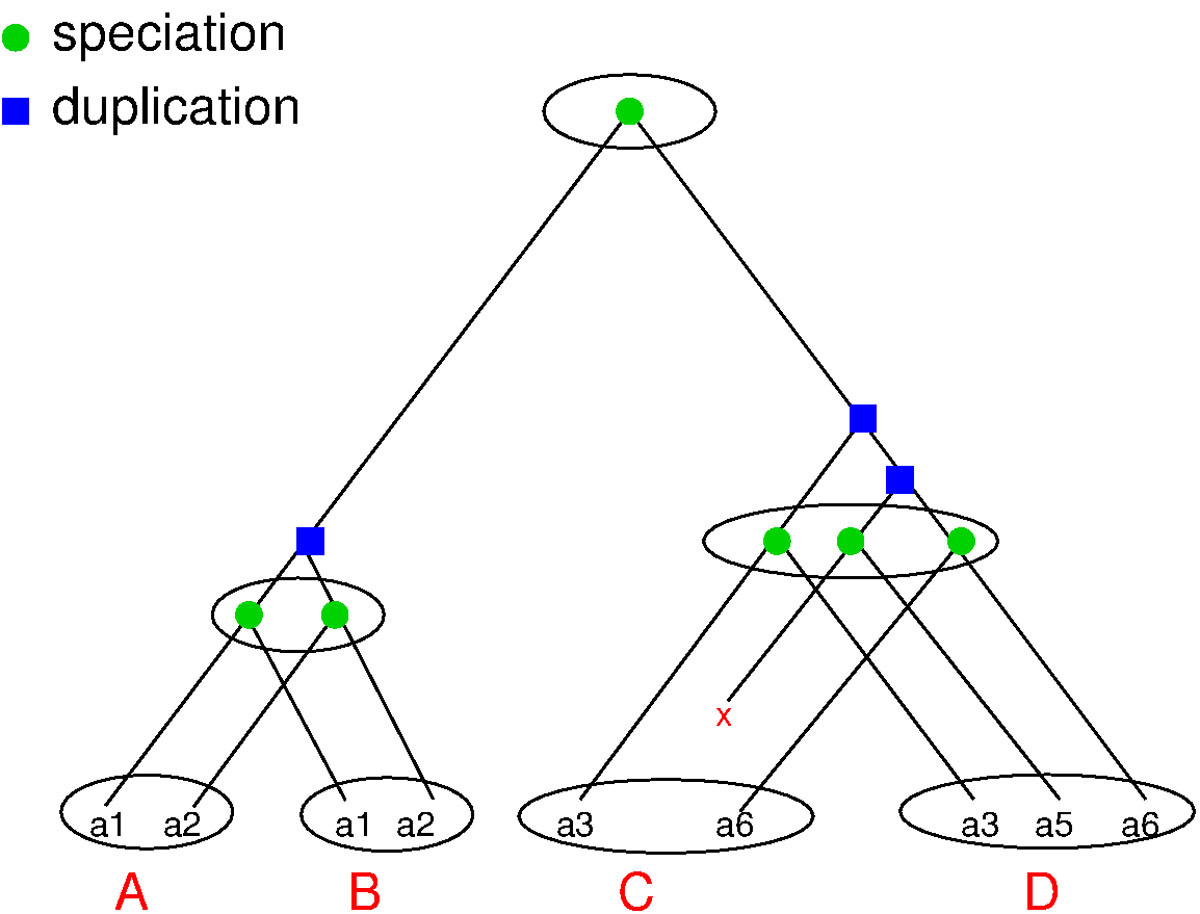
The reconciled tree: the gene tree embedded in the species tree. Each internal node represents an event, either a speciation or a gene duplication.

The user will introduce n number of genes (gene families), which will be placed at the root of the generated species tree T. T will then be traversed in a depth first order. For each visited edge a number of events is sampled from a stochastic Poisson Process Pλ, l where  λ is the probability of the event to happen and l the branch length. The process may generate none, one or a series of these events: one gene gets duplicated (gene duplication), a group of genes gets duplicated (cluster duplication), the whole group of genes gets duplicated (genome duplication) and one gene of the species gets lost (gene loss). After each gene duplication, one of the copies will be lost with a user defined probability θ, based on the fact that when there is a gene duplication, one of the copies might be lost or become nonfunctional. In the case of a cluster or genome duplication, we apply this probability to every gene in the group, since it is known that in the wake of multiple gene duplications and in particular for genome duplications we have to expect that many duplicated genes are rapidly lost again through the formation of pseudogenes. A small example of a gene family history generated by our simulation is shown in Fig. 1. We also show the gene tree generated from the gene family history embedded in the species tree. Each leaf node represents a gene and each internal node represents an event (speciation or duplication). This tree is typically depicted as the reconciled tree as in Fig. 2.

Finally, the algorithm will generate one gene tree for each species, i.e. the pruned reconciled tree containing only genes of a certain species. Furthermore, for each gene family the orthology and homology matrices are computed. To generate the orthology matrix, we say that two genes are orthologous if their lowest common ancestor (LCA) in the reconciled tree represents a speciation event. To generate the homology matrix, a gene a from species i is homologous to gene b from species j if for every gene c from species i and every gene d from species j the LCA(a, b) ≤ LCA(c, b) and LCA(a, b) ≤ LCA(a, d).

## Conclusions

We propose an algorithm that simulates gene family histories akin to real data. This will allow reconstruction algorithms to measure their accuracy and performance. Given a certain reconstruction method one might ask if the orthology matrix could be deduced from the inferred reconciled tree or if the homology relation between the genes was predicted correctly. Furthermore it could be analysed if the method was able to infer the gene duplications and losses. A method that is able to detect large scale duplications will then identify the cluster and genome duplications generated by our algorithm.
